# Role of Patient’s Ethnicity in Seeking Preventive Dental Services at the Community Health Centers of South-Central Texas: A Cross-Sectional Study

**DOI:** 10.3390/dj11020032

**Published:** 2023-01-28

**Authors:** Girish Suresh Shelke, Rochisha Singh Marwaha, Pankil Shah, Suman Challa

**Affiliations:** 1 School of Dentistry, University of Texas. Health Science Center San Antonio, San Antonio, TX 78229, USA; 2 The Helping Restore Ability, Arlington, TX 76018, USA; 3 Department of Urology, University of Texas. Health Science Center San Antonio, San Antonio, TX 78229, USA

**Keywords:** rural dental care, preventive visits, rural vs. urban

## Abstract

Background: This study was conducted to determine the impact of a patient’s ethnicity on seeking preventive dental services at the Community Health Centers (CHCs) in South-Central Texas. Methods: Primary electronic health records (EHR) data were collected regarding each patient’s medical and dental history, and comprehensive treatment planning. The researchers retrieved EHR from January 2016 to 2022. Bivariate analysis was completed to test the outcome with the predictor variable and covariates using the appropriate statistical tests. A multiple linear regression model was used to understand the association between the predictor and outcome variable while controlling for confounders. Results: The study findings revealed significantly higher dental visits (2.26 ± 2.88) for Hispanic patients. The results from the multiple regression model indicated that non-Hispanic patients had a smaller chance of visiting CHC for preventive dental services, by eight percent, compared to the Hispanic population (*p*-value < 0.001) when all other variables were held constant. However, the study results were not significant, as the effect size was too small to conclude the effect of ethnicity on the patients visiting the dental clinic at the CHC for preventive services. Conclusion: The study concluded that there is no difference in the preventive dental services completed by Hispanics and non-Hispanics when all other variables are controlled.

## 1. Introduction

According to the American Community Survey’s data from 2016, while the rural population constitutes only 19 percent (61 million) of the total population in the United States, it is spread across 97 percent of the land area. Trends suggest that the population in the rural areas is increasing as the majority of baby boomers retire and move to these areas [1]. In 2018, South-Central Texas, including Gonzales, Caldwell, Bastrop, Guadalupe, and Victoria, had a population of 405,558 [2]. Most individuals living in rural areas access community health centers (CHCs), and rural CHCs encounter more challenges in providing oral health care compared to urban CHCs [3]. The previous studies indicated that the patients in the rural areas visited the CHCs for dental services less frequently compared to urban centers [3] due to barriers such as income, age, and public insurance.

The individuals living in rural areas have limited access to oral and dental care, which is interlinked with other sociodemographic characteristics such as age, employment status, income, and insurance status [1]. Although the dental insurance coverage is significantly higher for adults in the age group of 20 to 64 years, they have the least number of dental visits (39.45%) compared to the pediatric (19 years and younger) (47.95%), and geriatric (65 years and older) populations (46.72%) [4]. Additionally, the Hispanic population reported fewer dental visits to dental clinics (33.6%) compared to the non-Hispanic White population (46.8%) [4]. The unfavorable sociodemographic factors such as lower income, lack of insurance, older age, and ethnic beliefs about health care reduce the dental care utilization at the community health centers in rural areas. This study focused on understanding the sociodemographic barriers in the oral health care utilization for preventive dental services in the Hispanic population.

Recent data suggests that by 2050, the proportion of older adults in the United States will increase from 54.1 million to 83.7 million. According to the Medical Examination Panel Survey (MEPS), adults above the age of 64 (27.52%) visit less for preventive dental services compared to the age group of 18 to 64 (30.17%) [4]. Most older adults choose to live in rural areas, which could potentially increase the burden of oral health diseases if dental utilization is low [5]. The 2018 report on the burden of oral health diseases in Texas suggests that access to oral health care in the rural population is insufficient as there are challenges to access oral health care due to unfavorable sociodemographic factors and distance from the health care facilities in the rural areas [2]. Additionally, the dentist-to-population ratio in rural South-Central Texas is 1:4143, which is 50% less than other urban counties in the region, indicating a disparity in the oral health workforce in these areas [2].

The nation’s first community health center (CHC) was opened in 1965 to provide affordable health care options for more than 29 million Americans [6]. The CHCs across the nation attempted to reduce barriers to primary and dental care by providing the necessary services to people, including indigent, uninsured, and underserved populations [6]. In Texas, 72 CHCs serve a population through 660 service delivery sites [7]. Though CHCs are the primary source of dental care in rural areas, they grappled with challenges such as lack of funding, distance, and cultural barriers in specific ethnicities [7]. The CHCs primarily serve patients who are using public insurance such as Medicare and Medicaid. Medicare is federal health insurance for people who are 65 or older, and some people who are under 65 and have certain disabilities or conditions. Medicaid is a joint federal and state program that helps cover medical costs for some people with limited income and resources [8].

There is substantial progress in improving the quality of oral health life, though there are predominant disparities in ethnicity, age, insurance, and income in the rural areas of the United States [9,10]. Additionally, ethnicity is closely related to dental services utilization and individuals’ total number of dental visits. According to the MEPS data, the Hispanic population reported fewer dental visits to dental clinics (33.6%) than the non-Hispanic Whites, i.e., 48.6% [9]. The current U.S. federal policy resulted in substantive dental health care improvement, but the disparities according to ethnicity are still significant [11,12] The 2009–2012 National Health and Nutrition Examination Survey (NHANES) reported that periodontal disease prevalence increased with age and is about 20% less amongst non-Hispanic Whites than Hispanics and Blacks [10].

Data from the 2015 Oral Health and Well-Being survey of the American Dental Association’s (ADA) Health Policy Institute reflects that the cost of dental services is 2.7 times more likely to be a reason for not visiting a dentist compared to factors such as fear and access [9]. The higher cost of dental care is the top barrier in dental care utilization (59%) regardless of age, dental insurance, or income level [9]. Among American adults, dental insurance coverage (71.4%) is approximately 20% less than health insurance coverage (92%) and reported the least (38%) among older adults (65 years and over) [4,5,6]. The Healthy People 2030 initiative aims to improve dental insurance coverage from 54.4% to 59.8%, as reported by the National Health Interview Survey (NHIS) [4,11].

The total number of dental visits in the income group that is 400% above the federal poverty level (FPL) is 27.2% more than the income group below 100% FPL [9]. The data collected for 2015 by the Medical Expenditure Panel Survey (MEPS) reflects that people with a college education are twice more likely to visit a dental clinic (50.59%) compared to people with no college education (25.38%) [9]. All these sociodemographic factors lead to a lack of access to oral health care, which is further affected by the low dentist-to-population ratio in rural areas [2].

Most patients reported to the dental clinic at the community health centers (CHCs) when they were in pain or needed emergency treatment rather than utilizing comprehensive dental treatment on a regular basis [13]. The data collected in 2015 by the Medical Expenditure Panel Survey (MEPS) reflects that approximately 138 million people in the United States visited the dentist at least once and collectively received services for 589 million procedures [14]. Out of 598 million procedures, preventive services only accounted for 33.96%. It is proven that oral health diseases such as dental caries and periodontal disease can be prevented by maintaining oral hygiene and regular dental visits, but there are profound gaps in access to preventive oral health care [14]. Patients are more likely to receive restorative and rehabilitative treatment rather than preventative dental services [14].

The previous studies identify the impact of ethnicity, age, insurance, and income on seeking oral health care and utilization of dental services at a national level; however, there is a dearth of information on how these sociodemographic factors influence the utilization of dental services, especially preventive care in rural areas [9,13,14]. This study aims to identify the influence of sociodemographic factors on seeking preventive dental care by individuals visiting the CHC in South-Central Texas.

The South-Central Texas population shows a higher percentage of the Hispanic population. The general population is predominantly divided into Hispanic and non-Hispanic groups. The Hispanic population includes undocumented immigrants with no insurance coverage, as well as most of the Hispanic ethnicity group, belonging to the lower socioeconomic class [7]. The primary objective of this research was to assess the role of patients’ ethnicity in the utilization of preventive dental services (oral examination, diagnostic radiographs, dental sealants, oral prophylaxis, and fluoride treatment, etc.) at the community health centers (CHCs) in South-Central Texas from January 2016 to January 2022. The study hypothesized that patients of Hispanic ethnicity were less likely to visit the dental clinic at the CHC in South-Central Texas to receive preventive dental services compared to non-Hispanic patients

## 2. Materials and Methods

This research was deemed exempt (protocol number: 20210881NHR) by the Institutional Review Board (IRB) at UT Health San Antonio. Following IRB approval, our research team collaborated with the information technology (IT) team from the South-Central Texas community health centers (CHCs) to retrieve data for this study. The CHC in South-Central Texas provide healthcare services to six counties at nine locations. The healthcare services include dental, family medicine, women’s health, pediatrics, behavioral health, and diagnostic laboratories. Additionally, patients do not have to be insured to receive the care at this CHC, and all patients without health insurance are evaluated during their first appointment to determine if they qualify for the sliding fee discount program as well as the public assistance program.

The CHC team uses the Greenway Health-Mediadent system to collect patient information [14]. The CHC records each patient’s medical and dental history, sociodemographic factors, dental diagnosis, and treatment plan after receiving the patient’s written informed consent. Each patient’s dental records are electronically integrated into their medical records, and the data are finally retrieved from the Intergy software, which is a data integration and storage platform of the Greenway Health–Mediadent system [15].

In this research, the confidentiality of protected health information (PHI) was a prime concern, so limited access was provided to the research team. The CHC information technology team de-identified the electronic health record (EHR) data and provided data from clinical dental reports for patients visiting the CHC from January 2016 to 2022. All other patient data before January 2016, including data for homeless patients, was excluded from the study.

**Figure A1:**
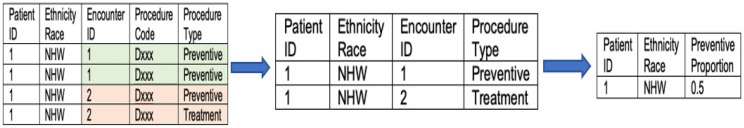
Data merging to convert the patient’s clinical encounters to patient visits.

Datasets for this cross-sectional study were cleaned and re-created using variables of interest, including gender, age, ethnicity, income, public insurance status, and the patient’s visits related to preventive dental services from the CHC’s dental clinic data. The data from the CHC includes encounter-level data with 202,354 observations for the variables of interest. Clinical data were recoded for each patient visit. Hence, there were data from multiple encounters for each patient, and each patient encounter included data on the dental services provided during that visit. A single row should represent each patient visit in the dataset to satisfy the independence assumption of the regression analysis as determined in the statistical analysis plan. Data were re-coded for all dental procedure codes, which denote the dental services completed during the patient encounters. Data were then recategorized based on the type of dental services provided as either preventive or treatment-related visits. The dental visits which only reflected the completion of preventive dental procedures such as scaling and root planning, fluoride application, and prophylaxis were classified as the preventive dental procedures if there were no other treatment procedures recorded. If the patient completed at least one treatment service during a specific dental visit, it was classified as the visit related to the treatment, even if a patient had any services related to prevention during that same visit. This is because the visit was primarily motivated by treatment services. Subsequently, the total number of visits to the CHC, preventive dental service visits, and preventive visits proportion were computed from the procedure codes. The final observations after merging the data on various patient levels were reduced to 21,774 (Figure A1).

The Ethnicity Variable: The ethnicity variable is binary and divided into Hispanic and non-Hispanic groups. The non-Hispanic ethnicity include all the races which are non-Hispanic including Black, White, Asian, Native American, etc.

We did not calculate the sample size and we included all the eligible patient data from the community health center after following both inclusion and exclusion criteria. The random sample was selected to represent the population served by the community health center in the South-Central Texas region. The larger sample size on the patient encounter level data (202,354) and individual patient level data (21,774) reduced the sampling bias.

The statistical analyses were conducted using SPSS, version 28.00. The dependent variable for the analysis was the patient’s dental visit for receiving preventive dental services. The independent variable of interest was the patient’s ethnicity. Other covariates included age, income, and public insurance status. A univariate analysis was performed to explore frequencies for the variables of interest and covariates. The bivariate and correlation analysis were conducted to understand associations between the categorical and continuous predictor variable with the continuous outcome variable, i.e., patient dental visits for receiving preventive dental services. A multiple linear regression model was used to analyze the associations between the outcome and predictor variables with other covariates. Following this, the stepwise selection method was used for final model selection. The final model was interpreted to understand the effect of the predictor variable (patient ethnicity) on the outcome variable (dental preventive visits) while controlling for confounders.

## 3. Results

**Figure 1:**
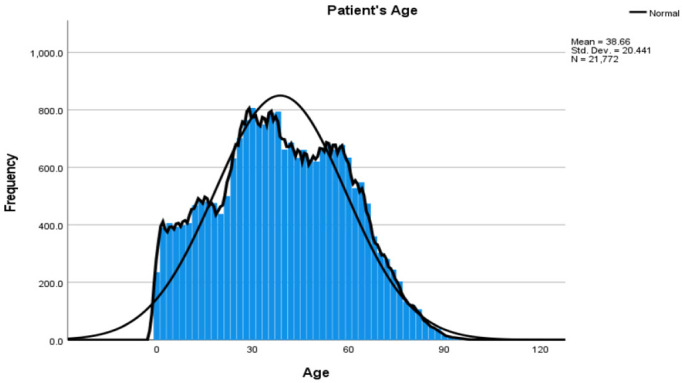
The Kernel Density Curve for Age.

After transforming the encounter level data to the patient level, the sample size for analysis was 21,774. The patient population visiting the community health center (CHC) varied by ethnicity, income, age, and insurance status. The mean age of the patients visiting the CHC was 38.66 years (±20.44) (Figure 1). The kernel density curve suggested that the curve was slightly right skewed with a median age of 38 years (Figure 1). The mean was slightly higher than the median for the age of patients visiting the CHC. More than half of the population visiting the CHC were Hispanic (52.9%). Very few patients had public insurance, reflecting only 21.8%. Most patients visiting the CHC reported an income of less than $25,000 (84.8%). The descriptive analysis results from Table 1 indicate that the mean dental visits related to the preventive dental services in a Hispanic population are 2.26 + 2.88 and the total dental visits in CHC for Hispanics were 4.62 + 4.86.

Several bivariate tests were conducted to understand the association between the dependent variable, preventive dental visits, and the independent variable, the patients’ ethnicity (Table 1). The differences in patient ethnicity and patients with public insurance were tested with the outcome variable using the independent sample T-test. This study found that patients who were Hispanic had significantly higher dental visits (2.26 ± 2.88 visits) compared to non-Hispanic patients (1.78 ± 2.33 visits, t (21,336) = 13.33, *p*-value < 0.001). Patients with public insurance who visited the CHC for preventive dental visits were significantly higher (2.68 ± 3.21 visits) compared to the patients with no public insurance (1.87 ± 2.44 visits, t (21,369) = 17.34, *p*-value < 0.001). Furthermore, a Pearson correlation test was used to determine the relationship between the patients’ ages and preventive dental visits at the CHCs, which revealed a positive correlation (r = −0.03, *p* < 0.001). A one-way ANOVA test was conducted to understand the differences between four income groups, which showed the statistical significance (F (621,363) = 25.83, *p*-value < 0.001). A Tukey post hoc test revealed that the preventive dental visits for the patients with an income less than $25 k were lower (2.03 ± 2.61 visits, *p* < 0.001) than the patients with income between $25 k and $45 k {(2.32 ± 3.06 visits, *p* < 0.001)} (Table 1).

The linear regression (Table 2) indicated that the outcome variable, dental preventive dental visits, is correlated with each patient’s ethnicity (R = 0.864), and the variation in each patient’s ethnicity is explained at least 74.6% (R2 = 74.6%) of the time. The overall regression model predicts the effect of independent variables on the dependent variable (*p* = 0.000). The multiple regression model suggests that non-Hispanic patients have 8% less of a chance of visiting the community health centers for preventive dental visits compared to the Hispanic population (*p* < 0.001), when all other variables, such as age, income, and public insurance, and the total number of dental visits were held constant. We expected the minimum change in the beta value (in the regression model) to be at least 15%. The change in the estimate was low (8%), with a confidence interval limit of 11% to 4% change (B = −0.08, 95% of B = −0.11, −0.04). The results showed a significant protective effect for the Hispanic ethnicity but did not fulfill our minimum threshold expectation for change in the estimates effect (Table 2).

## 4. Discussion

The rural population has limited access to care compared to the population of urban areas, which is influenced by the socioeconomic status, age, ethnicity, income, and insurance status of these individuals [16]. Our study focuses on understanding the sociodemographic barriers, specifically ethnicity, in utilizing the preventive dental services at the community health centers (CHCs) in rural Texas using electronic health records (EHR). The study successfully used EHR information to create a new dataset from the existing data to classify and analyze the preventive dental visits for the oral health care provided at the CHC.

The study achieved its objective to assess the role of patients’ ethnicity in the utilization of preventive dental services (oral exam, oral examination radiographs, sealants, and fluoride treatment). The probability of detecting the association between dependent and independent variables depended on the magnitude of association in the target population, i.e., change in the beta estimates in the regression model [17]. The result of the study shows that the change in the beta estimate in the regression model is very small (B = −0.08, 95% C.I. of B = −0.11, −0.04), which was insufficient to provide evidence against the null hypothesis (Table 2). Therefore, this study failed to reject the null hypothesis and determined that there is no difference based on patients’ ethnicity in the preventive dental visits in the population visiting the CHC in South-Central Texas.

The Medical Examination Panel Survey (MEPS) data showed that preventive dental service utilization increased exponentially in the Hispanic population, from 29.47% in 2006 to 34.99% in 2015 [9]. This trend is supported by our study, which showed that Hispanics visit the CHC for preventive dental services more frequently than non-Hispanic populations.

The results of our study support the previous studies, in that dental utilization improves as public insurance includes more people [4,8,18]. The mean preventive dental visits (2.68 + 3.21) and mean total dental visits (4.68 + 4.85) are highest in the people covered under public insurance, supporting the above findings. The results of the study contradict the MEPS findings, showing that the patients with an annual income of $25 k to $45 k visit CHCs more frequently for preventive dental services than other income groups (2.32 + 3.06) [9]. The highest income group, i.e., on an annual income of more than 65 k, visits the CHC less frequently (1.10 + 1.88), which might be due to the preference of this group to use a private clinic instead of CHCs.

Our study used electronic health records (EHR) to generate a dataset according to variables of interest and study goals. The EHR data are generally available easily through the health care software used for treatment planning in dental clinics. They are an excellent resource for understanding the population-level data and trends in health care. The procedure codes in the data included dental radiographs, which are significantly higher in numbers than any other procedure codes. According to American Dental Association (ADA) guidelines, radiographs should be classified as diagnostic or preventive services instead of treatment [19,20]. Due to the way the data were recorded in the EHR, the preventive codes increased significantly. Dental radiographs are mostly taken for all patients before the provision of preventive and treatment services but are classified only as preventive services. To address this issue, we reclassified the data on the patient-visit level. Suppose a patient visited the CHC and completed the three services related to prevention and one treatment service. In such a case, the visit is classified as a treatment visit, even though there are three preventive services carried out in the same visit; this is because the dental visit’s primary purpose was the treatment, not the prevention. If a patient visits the dental clinic only for the preventive services, and no other treatment-related services were completed, it is classified as a preventive dental service, as the visit was solely motivated by prevention. This method reduced the misclassification bias and provided a framework for future studies [21] (Figure 1). We reclassify the raw data variables into preventive, total dental visits, and preventive proportion. This study provides the guidelines for creatively using the preventive visit records to analyze the population trends (Figure 1).

The important strength of the study is that it analyzes the data from the last six years, which provides a large sample size of the patients (21,774), using 202,354 patient encounters. The large sample not only reduces bias but also improves the validity and generalizability of the results [22]. This study uses a sample from rural Texas, representing the population of Texas and neighboring states. The study results can be applied to southern states with a similar Hispanic to non-Hispanic population ratio [23]. The study’s external validity is reasonable, as the results are applicable to the United States rural population [23]. Though this was a cross-sectional study, the study attempted to reduce the sampling, information, and misclassification bias to improve internal validity [22]. Moreover, the CHC is one of the primary health care facilities in the region; hence, the data analyzed represent the county population who visited the clinic for oral health care, which in turn further strengthened the internal validity of the study.

Despite several strengths, the study has some limitations. First, causality and temporality cannot be inferred due to the cross-sectional study design [24,25]. The study results denote a small percentage of variation in the beta estimates, suggesting that an alternate study design should be used to test the hypothesis (Table 2). If we were to replicate the study, then we would choose the retrospective cohort study design as the group of patients visiting CHCs can be selected and followed for a specific time [25]. This will provide guidance in understanding the causal relationships of sociodemographic factors in using preventive dental services. The dental visits and preventive procedures should also be studied in other vulnerable populations like pregnant women, and other racial groups and their effect on the population [24].

The second most important limitation of this study is the classification of the independent variable, i.e., patient ethnicity. The non-Hispanic population included the Black, Asian, and Native American populations as well. The sociodemographic factors influence these categories as well. If we replicate the study, then we would analyze the impact of individual races on the outcome of preventive dental visits.

## 5. Conclusions

The study concluded that there is no difference in the preventive dental services completed by Hispanics and non-Hispanics when all other variables are controlled. The study results implied that patients with public insurance visited the CHC more frequently for preventive dental services. Additionally, the patients with an annual income between $25 k and $45 k reported the highest preventive dental services compared to other income groups.

The CHCs should focus on using the EHR data to analyze the barriers to receiving oral health care. These can be effective steps to establish public health research programs in community-based non-profit organizations in rural areas to help provide evidence-based oral health care and identify the social determinants of health. The results of this study will help advocate for improving access to oral health care in rural areas on state and federal platforms.

The rural and urban populations can be studied effectively by comparing the results of this study with community dental clinics in urban areas. The retrospective or prospective cohort study design should be used for further studies. Future studies should be focused on using mapping software such as a Geographic Information System (GIS) to analyze the impact of the distance from the clinic on dental care utilization in rural CHCs.

## Figures and Tables

**Table 1:** Sociodemographic and Dental Visit Information for Patients Visiting the Dental Clinic from January 2016 to January 2022.

Variables	Data	Preventive Dental Visits			Total Dental Visits		
	Characteristics						
		Mean	S.D.	Test Statistics /*p*-Value	Mean	S.D.	Test Statistics /*p*-Value
Patient Ethnicity							
Hispanic	11,517 (52.9%) 10,254 (47.1%) 3 (0.0%)	2.26	2.88	13.33 */ <0.001	4.62	4.86	12.07 */ <0.001
Non-Hispanic		1.78	2.33		3.87	4.27	
Missing							
Public Insurance	4748 (21.8%) 17,026 (78.2%)						
Yes		2.68	3.21	17.34 */ <0.001	4.68	4.85	6.93/ <0.001
No		1.87	2.44		4.15	4.53	
Income							
0 to $25 k	18,470 (84.8%) 2402 (11.0%) 610 (2.8%) 291 (1.3%)	2.03	2.61	25.83 † /<0.001	4.21	4.55	29.28 †/ <0.001
25 k to 45 k		2.32	3.06	<0.001	4.97	5.17	<0.001
45 k to 65 k		1.62	2.25	<0.001	3.69	3.82	<0.001
65 k+		1.10	1.88	<0.001	3.09	3.63	<0.001
Age (Continuous Variable)	21,772	38.66 (Mean)	20.44	−0.03 †† /<0.001	38.66	20.44	0.06 ††/ <0.001

**Note:** Table 1 represents the bivariate test for the different predictors using the different test statistics. Significant level *p* < 0.05. S.D., Standard Deviation, IQR = Interquartile Range. *. Independent sample T-test, †. F-test, ††, Pearson’s correlation coefficient.

**Table 2:** Understanding the Impact of the Patient Ethnicity on the Preventive Dental Visits Using the Multiple Linear Regression Model.

Dependent	Independent Variable	Adjusted	F Test Variables	B ** R^2^ (%)	*p* Value †	95% CI ^††^ of B
**Preventive Dental Visits**	**Constant**	**74.6**	**12,543**	**0.35**	**<0.001**	**(0.27, 0.44)**
	Patient ethnicity					
	Hispanic			Reference		
	Non-Hispanic			−0.08	<0.001	(−0.11, −0.04)
	Age of the patient at the first visit			−0.01	<0.001	(−0.01, −0.01)
	Income			−0.12	<0.001	(−0.15, −0.09)
	Have government insurance (Yes or No)					
	Yes			Reference		
	No			0.26	<0.001	(0.21, 0.31)
	Total patient encounters			0.52	0.00	(0.51, 0.52)

**Note:** Table 2 represents the final model for the analysis using a multiple linear regression model. **. Beta coefficient, †. *p*-value of the regression coefficients, ††. 95% Confidence Interval (C.I.) of Beta Coefficient.

## Data Availability

The data were received from the Community Health Center of South-Central Texas (CHCSCT- https://www.chcsct.com/ (accessed on 08 May 2022). The IT team of the CHCST shared data as the secured data files with the research group at UT Health, San Antonio.
